# Automating the analysis of public saliency and attitudes toward biodiversity from digital media

**DOI:** 10.1111/cobi.70217

**Published:** 2026-01-18

**Authors:** Noah Giebink, Amrita Gupta, Diogo Veríssimo, Charlotte H. Chang, Tony Chang, Angela Brennan, Brett G. Dickson, Alex Bowmer, Jonathan Baillie

**Affiliations:** ^1^ Conservation Science Partners, Inc. Truckee California USA; ^2^ On the Edge London UK; ^3^ Department of Biology, Environmental Analysis Program Pomona College Claremont California USA

**Keywords:** conservation culturomics, conservation social science, environmental social media, folk taxonomy, natural language processing, zero‐shot text classification, ciencias sociales de la conservación, clasificación de texto de disparo cero, culturomía de la conservación, procesamiento del lenguaje natural, taxonomía popular, 保护社会科学, 保护文化组学, 环境社交媒体, 自然语言处理, 零样本文本分类, 民间分类法

## Abstract

Measuring public attitudes toward wildlife provides crucial insights into human relationships with nature and helps monitor progress toward Global Biodiversity Framework targets. Yet, conducting such assessments at a global scale presents challenges. Digital news and social media offer a rich record of public discourse, but extracting information about attitudes toward wildlife from these sources is not straightforward. Selecting effective search terms is complicated by differences between everyday names for taxa and their scientific or formal common names, and raw news and social media data are often cluttered with irrelevant content and syndicated articles. To address search term selection, we used a folk taxonomy approach that derives recognizable species groupings from shared common name endings. We identified syndicated articles by using cosine similarity on term frequency‐inverse document frequency vectors. To filter out irrelevant content while minimizing the need for corpus‐specific annotation and model training, we developed a 2‐stage relevance filter that uses unsupervised learning to reveal common topics and an open‐source zero‐shot large language model (LLM) to assign topics to article titles and estimate relevance. We conducted sentiment, topic, and volume analyses on the resulting data. To illustrate our method, we examined news and X posts containing search terms for bats, pangolins, elephants, and gorillas from 2019 through 2021, a period that covers the onset of the COVID‐19 pandemic. Up to 62% of articles containing bat search terms were unrelated to bats as wildlife, underscoring the importance of relevance filtering. News articles mentioning horseshoe bats, initially implicated in the outbreak, increased significantly in January 2020, with significant sentiment shifts in news and X posts mentioning horseshoe bats emerging later (October 2020). Our methods provide a practical application of modern, general‐purpose natural language processing (NLP) tools, including LLMs, for analyzing public perceptions of biodiversity relative to current events or conservation outreach and marketing campaigns.

## Abstract (es)

Automatización del análisis de las actitudes y notabilidad pública hacia la biodiversidad en redes sociales

**Resumen:**

Medir la actitud del público hacia la fauna silvestre brinda información crucial sobre las relaciones humanas con la naturaleza y ayuda a supervisar el progreso hacia los objetivos del Marco Mundial para la Diversidad Biológica. Sin embargo, es un reto realizar estas evaluaciones a escala mundial. Las noticias digitales y las redes sociales ofrecen un rico registro del discurso público, pero extraer información sobre la actitud hacia la fauna silvestre de estas fuentes no es sencillo. La selección de términos de búsqueda eficaces es complicada debido a las diferencias entre los nombres populares de los taxones y sus nombres científicos o comunes formales, sumado a los datos sin procesar de las noticias y las redes sociales que suelen estar repletos de contenido irrelevante y artículos sindicados. Para abordar la selección de términos de búsqueda, utilizamos un enfoque de taxonomía popular que deriva agrupaciones de especies reconocibles a partir de terminaciones comunes compartidas en los nombres. Identificamos los artículos sindicados utilizando la similitud coseno en vectores de frecuencia de términos e inversa de frecuencia de documentos. Para filtrar el contenido irrelevante y minimizar la necesidad de anotaciones específicas del corpus y el entrenamiento de modelos, desarrollamos un filtro de relevancia de dos etapas que utiliza el aprendizaje no supervisado para revelar temas comunes y un modelo de grande lenguaje (MGL) de código abierto y sin entrenamiento previo para asignar temas a los títulos de los artículos y estimar su relevancia. También realizamos un análisis de sentimiento, tema y volumen sobre los datos resultantes. Para ilustrar nuestro método, examinamos noticias y publicaciones X que contenían términos de búsqueda relacionados con murciélagos, pangolines, elefantes y gorilas desde 2019 hasta 2021, un período que abarca el inicio de la pandemia de COVID‐19. Hasta el 62% de los artículos que contenían términos de búsqueda relacionados con murciélagos no tenían relación con los murciélagos como fauna silvestre, lo que subraya la importancia del filtrado por relevancia. Los artículos de noticias que mencionaban a los murciélagos de herradura, inicialmente implicados en el brote, aumentaron significativamente en enero de 2020, con cambios significativos en la opinión en las noticias y publicaciones de X que mencionaban a los murciélagos de herradura que surgieron más tarde (octubre de 2020). Nuestros métodos proporcionan una aplicación práctica de las herramientas modernas de procesamiento del lenguaje natural (PLN) de uso general, incluidos los MGL, para analizar la percepción pública de la biodiversidad en relación con los acontecimientos actuales o las campañas de divulgación y mercadotecnia sobre la conservación.

## 自动化分析数字媒体中公众对生物多样性的关注度与态度

**【摘要】** 评价公众对野生动物的态度, 能为人类与自然的关系提供重要见解, 并有助于监测《全球生物多样性框架》目标的进展。然而, 在全球范围内开展此类评估, 仍面临诸多挑战。数字新闻与社交媒体虽提供了丰富的公众舆论记录, 但从中提取关于对野生动物态度的信息并不简单。物种的日常用名与其学名或正式通用名之间的差异导致有效检索词的选择十分复杂, 且原始新闻与社交媒体数据常充斥着无关内容和同步转载的文章。为解决检索词选择的问题, 我们采用了民间分类法, 利用物种的通用名结尾来整合可识别的物种群组。我们还通过术语频率‐逆文档频率向量间的余弦相似度计算, 识别出了转载文章。为过滤无关内容同时减少语料标注与模型训练需求, 我们开发了一个两级相关性过滤器, 利用无监督学习揭示共同主题, 并利用开源的零样本大型语言模型(LLM)为文章标题分配主题并评估相关性。我们对处理后的数据进行了情感、主题及数量分析。为验证方法有效性, 我们分析了2019至2021年间(覆盖新冠疫情爆发初期)包含蝙蝠、穿山甲、大象和猩猩检索词的新闻及X平台帖文。结果显示, 高达62%的蝙蝠检索词相关文章与野生蝙蝠无关, 这突显了相关性过滤的重要性。提及菊头蝠(曾被怀疑与疫情爆发有关)的新闻报道在2020年1月显著增加, 而相关新闻及X平台帖文反映的情感则在稍晚时期(2020年10月)出现变化。我们的研究方法为现代通用的自然语言处理工具(包括大型语言模型)提供了实际应用场景, 可用于分析公众对生物多样性流行事件或保护宣传与营销活动的看法。

## INTRODUCTION

Public interest in biodiversity is pivotal to the success of conservation efforts but varies significantly across species, geographies, and time. Although targeted conservation campaigns can amplify public engagement around focal species and catalyze policy change (Thaler et al., 2017), the systemic change needed to halt biodiversity loss requires cultivating public awareness and support for nature and biodiversity as a whole (Convention on Biological Diversity, 2022; Díaz et al., 2019). Monitoring public attitudes at scale is key to informing efforts to build this support because it reveals where engagement is low, how perceptions are shifting, and which strategies may resonate most effectively with diverse audiences.

Conservation culturomics—analyzing digital data to examine societal relationships with nature—offers a promising approach for understanding public interest in biodiversity (Correia et al., 2021; Ladle et al., 2016). Digital data sources offer global reach and cost‐efficiency over conventional opinion‐based surveys and can reveal information‐seeking behavior rather than self‐reported behavioral intent (Cooper et al., 2019). Although recent work has developed attention metrics based on Wikipedia page views (Millard et al., 2021; Vardi et al., 2021) and Google Trends (Burivalova et al., 2018; Cooper et al., 2019; Vardi et al., 2021), news and social media offer additional insights by contextualizing public attention to species (Roberge, 2014). News media narratives shape public perceptions (King et al., 2017), and social media platforms amplify viewpoints and foster discussion on issues, including biodiversity conservation (Chang et al., 2022; Papworth et al., 2015; Veríssimo, 2021). However, unlike Google Trends and Wikipedia page views, news and social media yield unstructured text data that require careful searching and filtering for relevant content.

Selecting effective search terms for species in keyword‐based search application programming interfaces (APIs) is a complex task, stemming partly from the mismatch between the specialized biological nomenclatures conservation experts use, such as scientific (e.g., *Rhinolophus affinis*) and specific common names (e.g., intermediate horseshoe bat), and the broader folk taxonomic terms the public uses (e.g., *bat* or *horseshoe bat*) that may encompass multiple related species (Beaudreau et al., 2011). This highlights a trade‐off between specificity and volume of relevant content when assessing public views on taxonomic groups. Using scientific (Jarić et al., 2020, 2016; Ladle et al., 2019) or full common names (Kulkarni & Di Minin, 2021; Roberge, 2014) as keywords enhances specificity but risks overlooking general references to species in folk taxonomies, potentially biasing search results toward scientific content, especially for species lacking well‐known common names. Conversely, common names for folk taxa are challenging to infer. Author‐defined common names of target taxa (e.g., Fink et al., 2020) have been used, but extending this approach to thousands of species is arduous and subjective. Additionally, some common names (e.g., *elephant*) appear as substrings within unrelated species names (e.g., *elephant seal*), requiring careful consideration when constructing search queries.

Another challenge in conservation culturomics is the use of species common names in nonbiological contexts, such as sports teams (e.g., Clemson Tigers), individuals (e.g., Tiger Woods), and other entities. Machine learning and natural language processing (NLP) approaches can be used to develop text classification models for filtering out such irrelevant results. These models predict whether a sample of text pertains to biodiversity conservation (Kulkarni & Di Minin, 2021), target species, or conservation topics (Egri et al., 2022; Hunter et al., 2023; Keh et al., 2023; Roll et al., 2018). However, they require extensive manual data annotation for training, are susceptible to biases in data labeling, and can have worse performance when applied in contexts that differ from those included in training.

To address these challenges, we developed a novel method to retrieve online news and X (formerly Twitter) posts about biological taxa of conservation interest that includes deriving a folk taxonomy from English common names via substring matching to simplify the identification of names used in everyday language to refer to animals. With this method, we sought to facilitate analyses of less well‐known species by grouping them into more broadly recognized taxa so as to overcome the limitations posed by the use of only scientific or full common names for these species. We also sought to identify spurious groupings of unrelated species in the derived folk taxonomies and correct them by incorporating negative search terms in API queries to enhance search specificity. We used a zero‐shot text classification model to filter out irrelevant content, a cutting‐edge machine learning approach that obviates the need for data annotation by generalizing to new tasks without additional training. We applied our method to searches of public discourse on several mammal taxa from 2019 to 2021, which encompasses periods before and after the United Nations World Health Organization officially declared the COVID‐19 pandemic on 11 March 2020. Early in the outbreak, interest in wildlife increased, particularly in potential zoonotic coronavirus sources, such as bats and pangolins (Petrovan et al., 2021; Vijay et al., 2021; Zhou et al., 2020). We explored changes in public perceptions toward bats and pangolins, which were implicated in the pandemic, and elephants and gorillas, which were not, by examining discourse volume and sentiment shifts over time.

## METHODS

**FIGURE 1:**
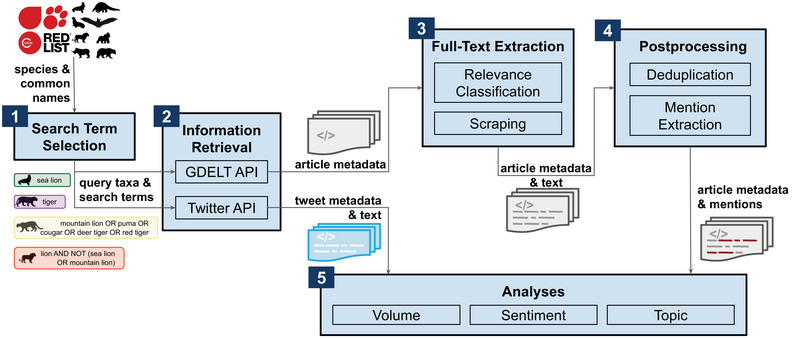
Steps in the automated workflow developed to analyze public salience and attitudes toward biodiversity in digital media: 1, construct a folk taxonomy to derive search terms; 2, retrieve news and social media posts by querying each data source; 3, perform zero‐shot relevance modeling and scraping to obtain the full text for the news articles; 4, filter out syndicated news and identify specific references to queried taxa in news articles; and 5, conduct analyses on shifts in volume, sentiment, and topics in posts and news articles over time and across geographies (GDELT, Global Database of Events, Language, and Tone; API, application programing interface; red list, International Union for Conservation of Nature Red List).

Our method for collecting online news articles and social media posts about biological taxa of interest (Figure 1) was composed of several successive steps. First, we selected taxa for analyses and obtained the corresponding search terms. Our focus was on taxa spanning a range of public visibility. This set the foundation for data collection by determining which species were encompassed in each targeted search. Next, we used keyword search APIs to retrieve online news articles and social media posts containing the specified search terms. We then classified article titles by topic to determine taxon relevance. Only relevant articles were selected for full‐text scraping. After scraping, we filtered out duplicate, often syndicated, articles and focused our analyses on sections of text referencing target taxa. Finally, we determined the volume of online content about different target taxa and how it varied geographically and over time. We used sentiment analyses to track shifts in the tone of these discussions and topic analysis to identify underlying themes in these discussions. We used these diverse approaches to deepen our understanding of the discourse dynamics related to the target taxa.

### Search term selection

Identifying salient folk taxa—species groupings that reflect how organisms are referenced in everyday language—is a fundamental step in understanding public perceptions of biodiversity. We focused on finding such groupings for mammals with English‐language common names. Species perceived as similar often share common name endings, and we applied this fact to identifying the groups of interest. To streamline the task of extracting candidate groupings from thousands of heterogeneous names, we developed a human‐in‐the‐loop approach that replaces the need for fully manual clustering with targeted expert review. Our approach involved 4 steps: identifying shared endings in common names, grouping species that shared each ending, organizing these groups into a hierarchy, and reviewing the hierarchy to select groupings that reflected public naming and familiarity.

We gathered the comprehensive list of 5650 mammalian species and their 9150 English common names from the International Union for Conservation of Nature (IUCN) Red List (IUCN, 2021).

**FIGURE 2:**
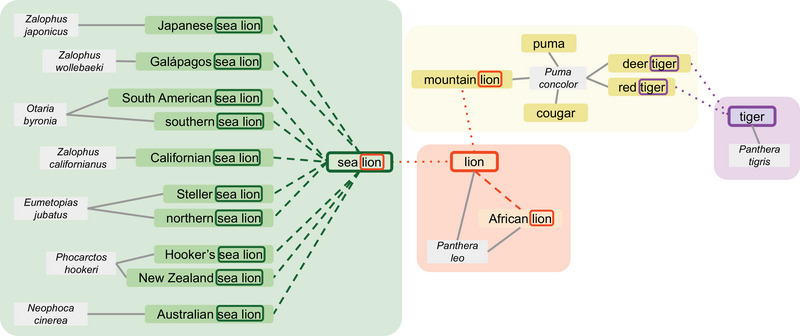
Example of an initial cluster of linked terms in the folk taxonomy graph for Carnivora species based on their International Union for Conservation of Nature Red List common names (solid lines, edges between species and their listed common names; dashed lines, edges between names and simplified names; dotted lines, connections that would be pruned on inspection to separate conceptually distinct taxa).

First, we identified the longest contiguous sequence of shared words at the end of each pair of common names (e.g., *sea lion* from *South American sea lion* and *Californian sea lion*) (Figure 2). We adapted the standard dynamic programming algorithm for the longest common substring problem (e.g., Gusfield, 1997) to compare space‐separated words rather than individual characters and retained only matches that occurred at the end of both names. Each unique shared name ending defined a prospective folk taxon: a set comprising all full common names that ended with that phrase (e.g., all full common names ending in *sea lion*). We then constructed a hierarchy by detecting immediate subset relationships between sets.

To make emerging groupings more interpretable, we represented the set relationships described above as an undirected graph. Nodes corresponded to either individual species or the sets of full common names defined in the previous step, and edges were added when one set was an immediate subset of another. We used a breadth‐first search to cluster the graph into connected components, each representing a top‐level candidate folk taxon based on name endings. Additional details about the graph construction are in Appendix S1.

We manually reviewed each connected component in the graph to assess whether the automatically generated clusters aligned with intuitive or recognizable categories of species. This review validated the coherence of each set (i.e., whether the shared name ending grouped conceptually related species) and was used to assess whether the level of specificity matched how species are typically distinguished in everyday language. When needed, we removed edges to separate distinct groups or merged or split components to reflect categories likely to be salient to a general audience. These adjustments informed the final construction of search keywords for each taxon, including the introduction of negative keywords to prevent conflation between unrelated taxa that share the same name ending.

For each folk taxonomic entity, we compiled a set of positive keywords, at least one of which had to be present in a search result, and an optional set of negative keywords, all of which had to be absent from a search result. We selected 10 focal taxa for analysis: elephant, gorilla, pangolin, bat, flying fox, myotis, horseshoe bat, pipistrelle, vampire bat, and long‐tongued bat. The corresponding scientific taxa and keyword sets for these focal taxa are provided in Appendix S2. These taxa were chosen to span a range of taxonomic specificity and public familiarity and allowed us to explore how these factors influenced the relevance and volume of retrieved content.

### News and social media information retrieval

We collected online news articles from the Global Database of Events, Language, and Tone (GDELT), a live database capturing global news media that offers full‐text searches via the GDELT 2.0 DOC API. Using positive and, where applicable, negative search keywords for each focal taxon, we requested English‐language articles published from 1 January 2019 to 31 December 2021. Each query returned JavaScript‐object‐notation–formatted article metadata that included the article's title, URL, domain, date, and country of publication. To work within the limit of 250 results per query imposed by the API, we divided the 3‐year period into shorter intervals, aggregating results from each interval to form our final dataset.

For social media analysis, we used Twitter Academic Access 2.0 API to access Twitter's full archive of public tweets. We queried this API with the positive and negative keywords for each focal folk taxon, requesting only tweets written in English and including geolocation data to support analyses on geographic differences in species media portrayals. Twitter data collection concluded before 9 February 2023, ahead of potential deprecation notices for the Academic Access API by Twitter.

### Relevance filtering of news items

Keyword‐based searches often retrieve content in which taxon names appear in nonbiological contexts. For example, the word *tiger* may appear in reference to sports teams (e.g., Clemson Tigers), public figures (e.g., Tiger Woods), companies (e.g., Tiger Global Management), places (e.g., Tiger Hill), or even events (e.g., Year of the Tiger). These results should be excluded from analyses focused on public attitudes toward the target taxa. The core challenge is to determine whether a given text was likely to use the taxon keywords that triggered its return in a biological sense based on the available textual information. Topic, in particular, provides useful cues about how a taxon name is likely to be used. Topics, such as conservation (Kulkarni & Di Minin, 2021), tourism, and outdoor recreation, are more likely to be associated with biological references to taxa, whereas other topics, such as sports or politics, are more likely to involve unrelated uses. This reasoning informed our relevance filtering for GDELT articles. We used the returned article title to predict topics prior to scraping. Articles were retained if any of the predicted topics were judged relevant to a biological use of an animal name.

Rather than manually creating a set of topics present in GDELT articles retrieved through taxonomic keyword search a priori, we used a data‐driven approach to induct a candidate topic set from article content. We queried GDELT for news articles from 2019 that used folk taxonomic search terms for mammalian species across 14 orders and randomly sampled 10,000 articles from the results to ensure that at least one taxon from each order was represented. The sample included 154 distinct query taxa. We obtained full‐text content via web scraping (see “Scraping full text of articles”) and extracted text snippets consisting of a sentence containing a search keyword and the preceding sentence to provide context. We applied latent Dirichlet allocation (LDA) (Blei et al., 2003), a common unsupervised topic modeling method used to reveal underlying thematic structure in large text corpora by modeling texts as mixtures of topics and topics as mixtures of words. After experimenting with different values, we specified 40 topics, which was sufficient to yield a diverse set of coherent topics while allowing the model to reliably converge within 150 iterations. We reviewed the 20 words with the highest probability of occurring for each topic to assign the topic a semantically meaningful label. We applied the same process to 2 additional GDELT corpora: one retrieved using search terms related to biodiversity threats and the other retrieved using terms related to global biomes (10,000 articles sampled in each case). These additional corpora were included to capture broader thematic contexts in which taxon names might appear. Uninterpretable or redundant topics were discarded or merged across the 3 models, resulting in a final set of 23 topics.

We classified each of the 23 topics as relevant or irrelevant based on whether they were likely to include taxon names used in a biological sense. N.G., C.C., A.B., and A.G. independently reviewed the labeled topics and resolved disagreements by consensus. The resulting set of relevant topics was intentionally permissive, encompassing outdoor recreation, tourism, agriculture, and infrastructure, contexts in which animal names may, but do not always, appear in a biological context. The topics of agriculture, climate change, conservation, energy, health, infrastructure, natural disasters, nature, outdoor recreation, science and technology, tourism, wildlife, habitat loss, invasive species, and pollution were classified as relevant. The topics of business, crime, education, entertainment, food, holidays, politics, and sports were classified as irrelevant.

After identifying a set of topics that captured the thematic contexts in which taxon names appeared, we classified article titles into one or more topics. We explored a multi‐label zero‐shot classification approach to assess whether a general‐purpose language model could assign topic labels to article titles without requiring labeled training data. Specifically, we used the bart‐large‐mnli variant of Facebook's Bidirectional and Autoregressive Transformers (BART) model (Lewis et al., 2020), a publicly available model trained on the Multi‐Genre Natural Language Inference dataset (Williams et al., 2018) and accessible via the Hugging Face transformers library. Trained on large and diverse text corpora, it learns numeric representations (embeddings) that capture semantic patterns in language and support generalization to new tasks. It performs well at zero‐shot text classification (Yin et al., 2019) by estimating how likely a given text supports class labels phrased as natural language statements (e.g., “This example is politics.”). Each article title received a score from 0 to 1 for each of the 23 topics listed above. If any of the relevant topics received a score >0.5, the article was flagged for full‐text scraping. This approach allowed us to triage large volumes of article titles for likely relevance before proceeding to scrape the article text.

To assess the performance of the topic‐based relevance filter, we conducted an evaluation on a stratified sample of 1000 articles. We sampled 100 articles for each of the 10 focal taxa included in our analyses and scraped their full text. We examined the text of each article and labeled it as either relevant or irrelevant. Comparing these ground‐truthed labels with the output of the relevance filter, we observed an overall precision of 83.6%, recall of 88.2%, and F1 score of 85.8%. Performance varied across taxa. Per‐class precision ranged from 26.2% to 100% and recall from 78.6% to 100%. Precision tended to be lower for general taxon names, such as *bat*, *gorilla*, and *elephant*, and higher for more specific names, such as *myotis*, *pangolin*, and *vampire bat*. Despite lower precision, the general taxa also had high negative predictive value (e.g., 94.8% for *bat*, 100% for *gorilla*, and 95% for *elephant*), meaning articles filtered out as irrelevant were almost always correctly excluded. This is especially useful given the high volume of articles for these taxa; downstream noise was reduced, and processing of irrelevant articles was avoided. Recall was consistently high across all 10 focal taxa, reflecting our choice to prioritize capturing relevant articles even at the cost of admitting some irrelevant ones. Manual review of false positives revealed that many misclassifications arose from the use of broad topic labels. Some terms, such as *energy*, *infrastructure*, and *outdoor recreation*, sometimes triggered high topic scores for articles that were only abstractly related to the topical label. Most false negatives involved articles in which the taxon was mentioned only incidentally or metaphorically, resulting in titles that did not strongly signal any relevant topic.

### Scraping full text of articles

To obtain the full text of news articles flagged as relevant, we first submitted an HTTP request for the HTML content of each relevant GDELT news article URL. If the request was successful, the HTML content was parsed using one of 3 Python libraries (trafilatura [Barbaresi, 2021], newsplease [Hamborg et al., 2017], or boilerpy3 [Riebold, 2024]) to extract the article body. Often, however, the HTML request or the text extraction was unsuccessful due to broken URLs. In these cases, we searched for a snapshot of the article on the Internet Archive. If a snapshot was found, we requested the HTML content of this snapshot and attempted to extract the article body text with the same combination of Python libraries as mentioned above.

### News data postprocessing

In mainstream media, news articles are often syndicated across multiple outlets with minimal changes to the text (Kulkarni & Di Minin, 2021). To prevent bias in downstream models and avoid redundant analyses on near‐identical content, we identified duplicates by measuring the similarity between articles. We used term frequency‐inverse document frequency to create a vector representation of each article's text based on its most distinctive words and then computed the cosine similarity between pairs of article vectors. We compared all pairs of articles published within 2 months of each other because syndicated articles are typically released soon after their originals. If the cosine similarity exceeded 0.95, indicating a high degree of similarity, we classified the later‐published article as a syndicate of the earlier one. Conversely, if an article's cosine similarity score with every other article published within the preceding 2 months was below 0.95, we classified it as an original.

Next, we isolated sentences in articles that directly referenced the target taxa, a step we called *entity mention detection*. This step enabled us to precisely apply NLP tasks, such as sentiment analysis and topic modeling, to text segments containing the entity of interest. This is especially useful for longer bodies of text (e.g., articles) that may discuss many different things and have shifts in tone. We scanned each article for the positive search terms for a target taxon. Upon finding a mention, we extracted the sentence that contained this reference along with the sentence immediately preceding it. Including the preceding sentence was helpful because it often provided additional context for the mention.

### Analyzing public discourse about species

For each focal taxon, we collected articles and tweets from 2019 through 2021. To determine the overall volume of discourse on each taxon, we aggregated the number of articles mentioning each taxon by month and country.

We examined the sentiment of media and public discussion of species with a lexicon sentiment model. Specifically, we used the Valence Aware Dictionary and sEntiment Reasoner (VADER) (Hutto & Gilbert, 2014). This yielded a sentiment score for each article ranging from −1 (strongly negative sentiment) to 1 (strongly positive sentiment). A score of 0 indicated neutral sentiment. We also aggregated these article‐level sentiment scores by month and country to explore trends in the tone of public discourse regarding species. Although VADER is widely used for social and news media (Chang et al., 2022; Fink et al., 2020), its general‐purpose lexicon can assign sentiment to conservation‐relevant terms (e.g., *threatened species*, *critical habitat*) in ways that do not always reflect domain‐specific usage (Lennox et al., 2020). In our case, sentiment analysis was a modular component of our method that could be replaced or extended with domain‐adapted methods.

We explored how conservation social science researchers and practitioners interested in messaging or marketing to conserve biodiversity can perform different analyses with the outputs of our data pipeline. Using information on the country where each news article is published, we generated choropleth maps to visualize the volume of public discourse about different taxa. We created chord diagrams to illustrate the distribution and co‐occurrence of topics associated with news coverage of each taxon. Finally, we applied breakpoint analysis (Killick & Eckley, 2014) to test for significant changes in the mean volume or sentiment over time for each focal taxon.

## RESULTS

### Folk taxonomy and data on target taxa

An expert‐guided manual review of graph components showed that the method produced mostly useful and interpretable groupings that captured broad distinctions and more detailed nested patterns. For example, the terms *rock wallaby* and *forest wallaby* emerged as distinct subsets within a broader *wallaby* grouping. The method also successfully grouped distinct regional common names for the same species, as in cases where names, such as *dassie* and *hyrax*, were linked through shared species associations despite having no overlapping name structure.

Shared name endings occasionally led to the conflation of distinct taxa (Figure 2). One connected component from Carnivora grouped several species under the folk taxon *sea lion*, a term the public is more likely to use than any individual species name (Figure 2). However, the same component also linked sea lions to *Panthera leo* (*lion*) and *Puma concolor* (*mountain lion*) due to the shared name ending *lion*. To avoid mixed search results for *lion*, we removed these edges—a decision that directly informed our use of negative keywords (e.g., *lion* and not *mountain lion* and not *sea lion*) to improve search specificity. This approach was distinct from filtering out articles that used terms, such as *lion*, in a nonbiological context (e.g., references to *Snoop Lion*), which were addressed by the relevance filtering step.

Related species were occasionally grouped or separated based on public familiarity and perceived distinctiveness. For instance, the node for *bear* grouped *black bear*, *polar bear*, and *sloth bear*, but given the widespread public recognition of different bear species, removing the *bear* node allowed searches to target each animal individually. In contrast, *woodchuck* and *marmot* appeared as separate clusters due to differing name endings and regionally distinct common names, but they were combined because the distinction was unlikely to meaningfully affect our analyses of online media.

**FIGURE 3:**
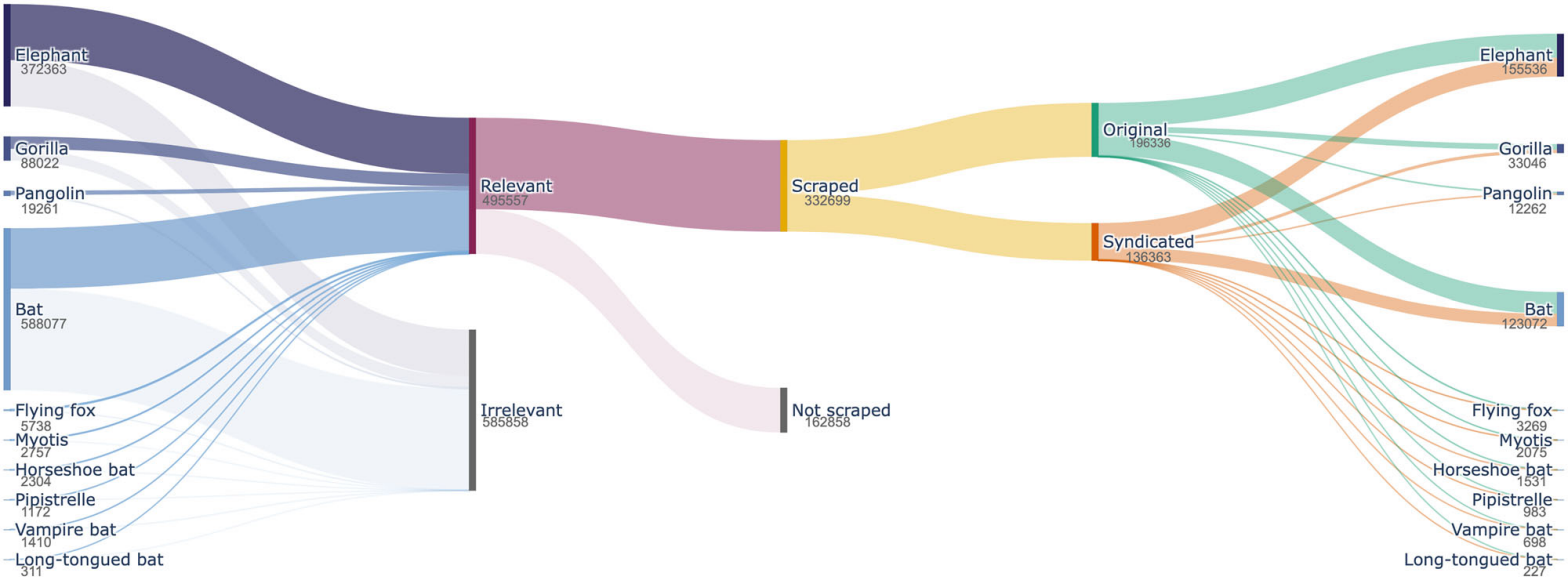
Number of news articles obtained at each stage (query, relevance filtering, web scraping, duplication elimination) in the news data collection process for 10 taxa.

Raw article counts varied from 588,077 for *bat* to 311 for *long‐tongued bat* (Figure 3). Approximately 54% of articles were predicted to be irrelevant to wildlife, with *bat* (62.6%), *gorilla* (48.4%), *elephant* (45.3%), and *vampire bat* (36.4%) yielding high proportions of unrelated content due to homonymy (e.g., *bat* as a piece of sports equipment), idiomatic expressions (e.g., “elephant in the room,” “800‐pound gorilla,” and “off the bat”), and popular culture depictions of these animals. One‐third of full‐text articles selected for scraping were inaccessible due to broken links. Forty‐one percent of articles across these taxa were syndicated, indicating significant potential for computational efficiency by limiting analyses to only relevant, original articles (Appendix S3). Ultimately, taxa with widespread popular appeal (elephants, gorillas) had more wildlife news articles than lesser‐known taxa (pangolins), and generic taxa had more articles than specific ones.

On social media, the public made several hundred to nearly 300,000 posts about different taxa from 2019 to 2022 (Appendix S4).

### Geographic variation in discourse

**FIGURE 4:**
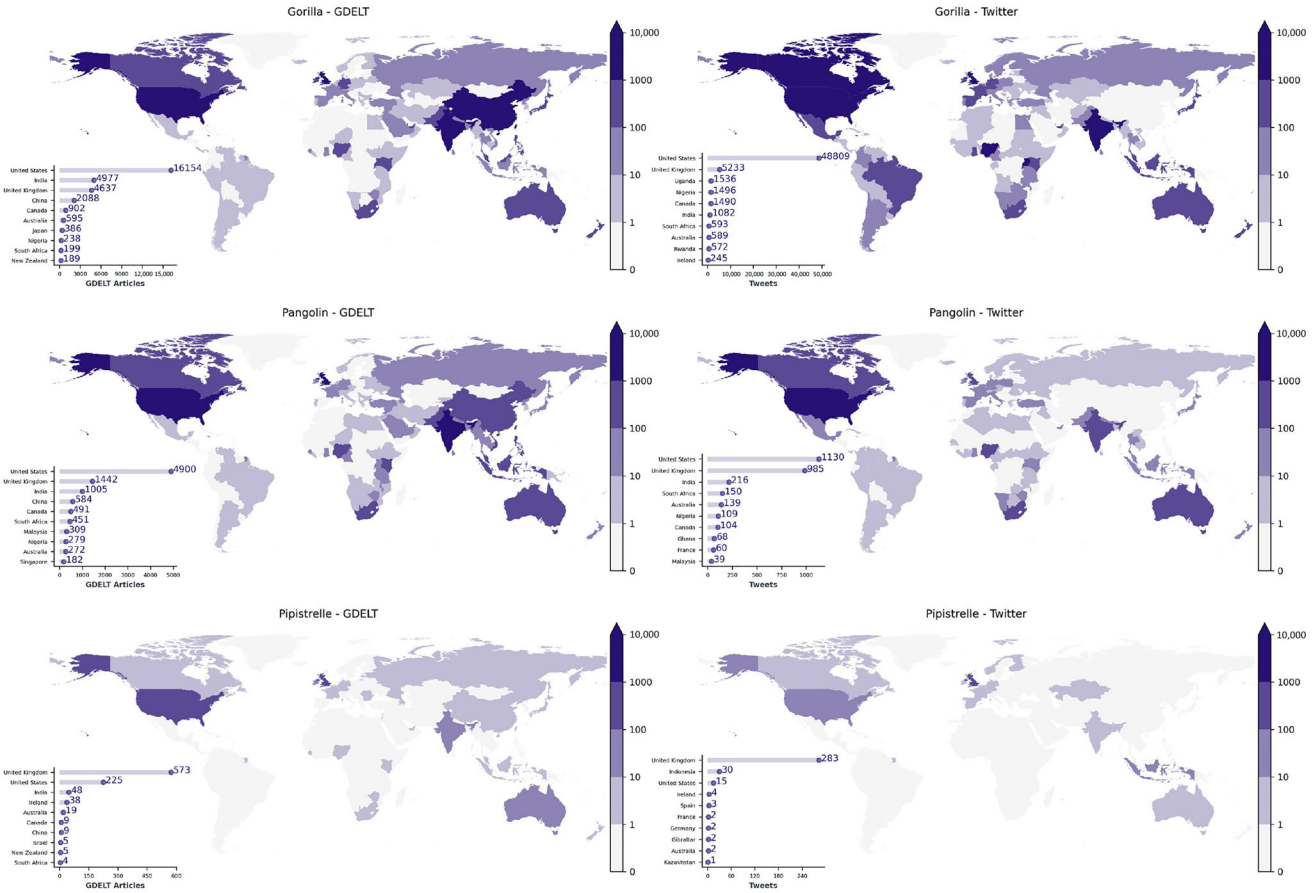
Volume of relevant news articles from the Global Database of Events, Language, and Tone (left) and Tweets (right) from 1 January 2019 to 31 December 2022 for gorillas, pangolins, and pipistrelles. Insets show top 10 countries by volume.

Globally recognized animals, such as gorillas, received widespread attention online, whereas less well‐known taxa, such as pangolins and pipistrelle bats, saw more geographically concentrated coverage (Figure 4). Pangolins were primarily featured in Southeast Asia, whereas pipistrelle bats, despite their prevalence in the British Isles and widespread distribution in Asia, attracted less media attention outside the United Kingdom. Thus, media exposure to different animal taxa varied by geography, potentially influencing levels of awareness and familiarity.

Beyond differences in overall coverage volume, sentiment varied across countries and platforms. Among the countries most frequently mentioning pangolins in news and Twitter, Singapore had more pangolin‐related news coverage than Ghana, and the average sentiment of those articles was more positive in Singapore than in Ghana (0.09 vs. −0.12). On Twitter, Ghana had a higher tweet volume about pangolins but a lower average sentiment score (0.13) compared with Singapore's smaller but more positive set of tweets (0.33). In Ghana, discourse tended to focus on poaching and trafficking, whereas in Singapore, coverage included trafficking but also emphasized pangolin sightings, legal protections, and public engagement. These contrasts illustrate how the same taxon can be embedded in distinct narrative contexts depending on regional conservation dynamics. Our method enabled this type of cross‐country comparison and offered a richer understanding of how biodiversity is framed across cultural and geographic settings.

### Topics associated with different taxa

Co‐occurrence of topics predicted by Facebook's BART model (Lewis et al., 2020) showed that topic distributions varied across taxa. Some taxa appeared primarily in a narrow set of topical contexts, and others were associated with a broader, more interconnected range of topics. Appendix S5 contains a full set of chord diagrams for all folk taxonomic entities.

**FIGURE 5:**
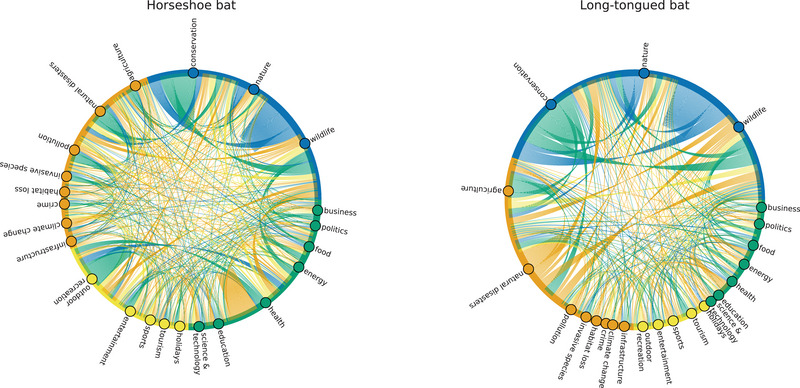
Co‐occurrence of topics in relevant news articles for horseshoe bat (*Rhinolophidae*) and long‐tongued bat (*Glossophaga*, *Craseonycteris*, and *Leptonycteris*) (arcs, topics occurring together in articles; the wider the arc, the more articles that contained a given topic pair; colors, different groups of topics; sections on the perimeter of circles, proportional occurrence of each topic [e.g., sports] in the dataset).

Comparing horseshoe bats, a known reservoir of SARS‐CoV, versus long‐tongued bats, which are not regarded as a coronavirus reservoir, the distribution and co‐occurrence of topics were quite different between these 2 groups of species (Figure 5). Long‐tongued bats had much more news coverage devoted to nature‐based topics, such as conservation or wildlife. Moreover, the nature or conservation threats topics (e.g., nature, wildlife, climate change, habitat loss, etc.) tended to co‐occur within an article. In contrast, horseshoe bat media coverage exhibited comparatively more discourse on the topics of conservation threats (e.g., habitat loss, natural disasters, climate change) and socioeconomic issues (e.g., business, health, education). Health and food were more prevalent topics for horseshoe bats compared with long‐tongued bats. For both types of bat, however, the chord diagrams indicated broadly distributed topical associations rather than a few dominant themes or strongly recurring topic pairs at the level of individual articles (Figure 5).

### Change in salience of taxa through time

**FIGURE 6:**
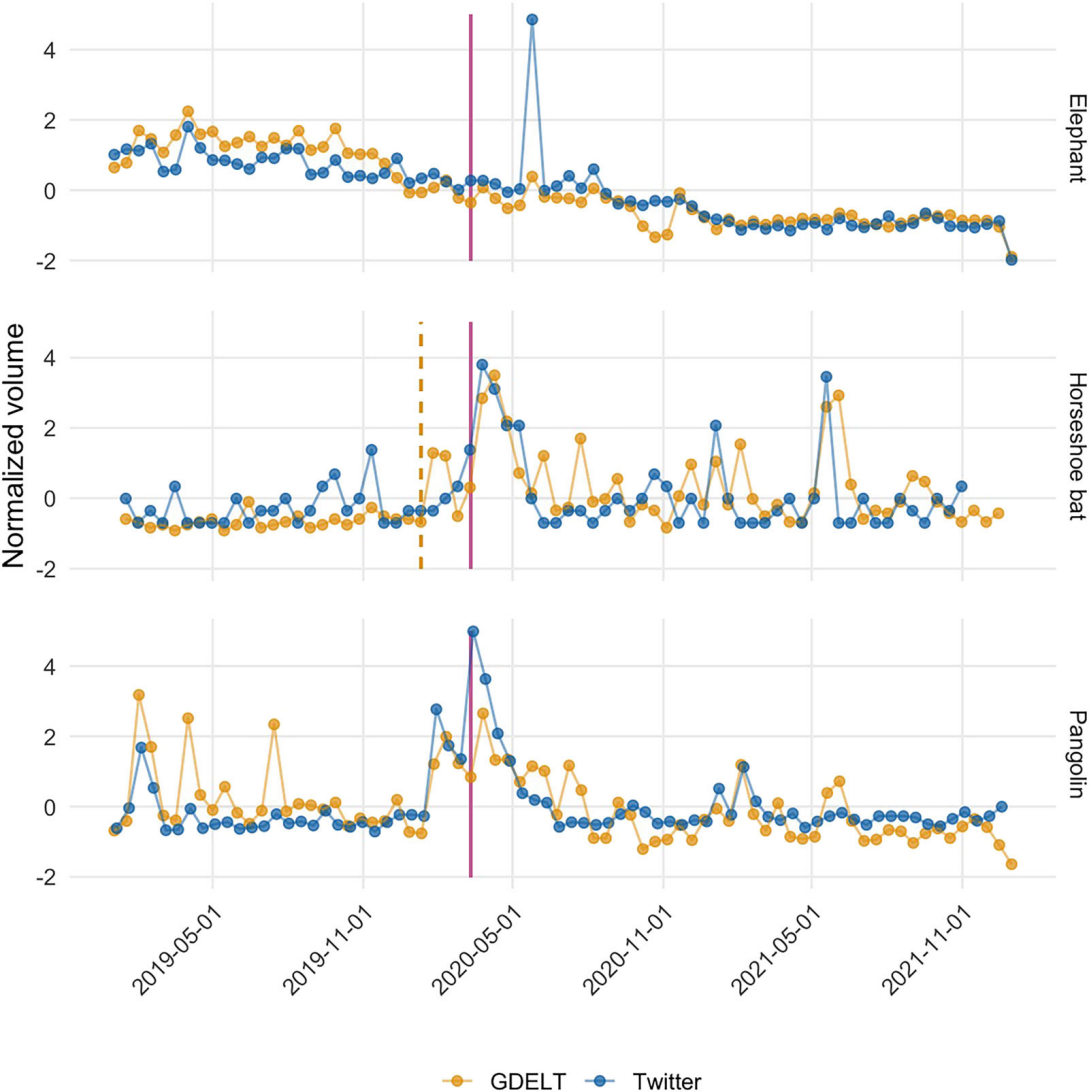
The *Z*‐score‐normalized time series of the number of news articles (GDELT, the Global Database of Events, Language, and Tone) and social media posts (Twitter) mentioning elephants, horseshoe bats, and pangolins (vertical magenta line, World Health Organization's COVID‐19 pandemic declaration on 11 March 2020; dashed vertical gold line, Bonferroni‐corrected significant breakpoint in the trend for news volume).

Changes in the volume of news articles and tweets referencing different taxa showed clear contrasts in overall trend, timing, and magnitude (Figure 6 ). The salience of taxa implicated as coronavirus hosts or potential spillover hosts (pangolin or horseshoe bat) and the salience of species of conservation concern that were not clearly associated with COVID‐19 (e.g., elephant) differed. A breakpoint analysis indicated a significant change in the volume of news media articles mentioning horseshoe bats, with an average of 3 articles every 2 weeks before 10 January 2020. This number jumped to an average of 20 articles every 2 weeks after. We found no other significant breakpoints for news article or tweet volume for pangolins or elephants, or for tweet volume for horseshoe bats (Figure 6). However, there were consistent annual increases in pangolin‐related articles and tweet volume around World Pangolin Day across all 3 years in our dataset. For elephants, we found a major spike in Twitter activity in early June 2020 related to an elephant mortality incident, but there was no comparable increase in news. For the full set of focal taxa (Appendix S6), we identified additional breakpoints in news media coverage for flying fox, gorilla, and myotis from November 2019 to September 2020, although, unlike horseshoe bat, these were associated with no change or a reduction in the average article volume. We did not find any significant breakpoints in tweet volume for any taxa.

**FIGURE 7:**
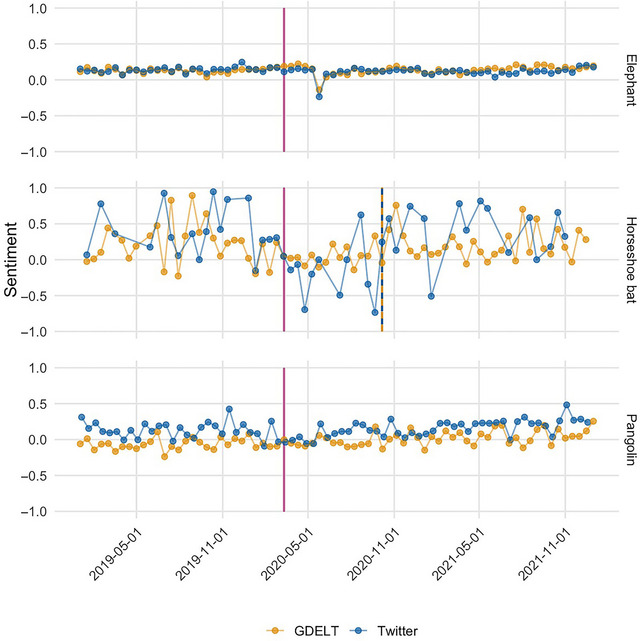
Time series of the sentiment of news articles (GDELT, the Global Database of Events, Language, and Tone) and social media posts (Twitter) mentioning elephants, horseshoe bats, and pangolins (vertical magenta line, the World Health Organization's COVID‐19 pandemic declaration on 11 March 2020; dashed vertical gold or blue lines, Bonferroni‐corrected significant breakpoints in the trends for news or social media, respectively).

### Change in discourse about taxa through time

We showed that monitoring of changes in public sentiment around different taxa can be conducted with the outputs of our pipeline. For elephants, horseshoe bats, and pangolins (Figure 7), the mean sentiment score for taxa was lowest for pangolins in the news (mean of −0.01) and highest for horseshoe bat on Twitter (mean of 0.28). Of these 3 taxa, pangolins in the news had the lowest average sentiment score but a higher sentiment score on Twitter (0.14), elephants had an average sentiment of 0.12 (news) or 0.13 (Twitter), and horseshoe bats had the highest average sentiment across the board (0.18 in the news and 0.28 on Twitter).

Across all the taxa we examined, only horseshoe bat had a significant breakpoint in sentiment score (Appendix S7). Horseshoe bat discourse showed a change in sentiment on 6 October 2020 in news media coverage and Twitter posts. The mean sentiment of horseshoe bat coverage remained the same (0.2) in the news media. However, Twitter horseshoe bat sentiment changed from an average of 0.2 to 0.4 (i.e., it became more positive through time).

## DISCUSSION

Our methodology allows practitioners and researchers to monitor public perceptions of biodiversity globally and with geographic or temporal disaggregation. This method builds on several recent advances that use NLP and machine learning approaches to process and analyze large, unstructured text data on biodiversity. Kulkarni and Di Minin (2021) devised a method to retrieve news articles that mentioned 585 species listed in Appendix I of the Convention on International Trade in Endangered Species of Wild Fauna and Flora (CITES). Egri et al. (2022) analyzed articles from the *Times of India* for instances of human–wildlife conflict associated with 15 species in West Bengal. We extended these bodies of work by simultaneously scraping data from the news media and social media; by creating a folk taxonomy to broaden the data sampled; by using cutting‐edge large language models (LLMs) to filter our data in an efficient, performant, and replicable fashion; and by using the Internet Archive as a fallback source when original news article links were no longer accessible.

One advance of our approach is the use of string algorithms and graph theory to generate a folk taxonomy that can serve as a practical basis for keyword‐based searches. Selecting search terms for species is not trivial. Scientific or full common names provide specificity, but they often miss broader public references to taxa, especially in informal or nonscientific contexts. Conversely, coming up with broader search terms to capture groups of similar species is subjective and difficult to do at scale due to the need to make upfront decisions about group boundaries. We showed that this process can be simplified by automatically generating candidate taxonomic groupings based on common name endings, which allows experts to conceptually review which groups to keep, split, or combine and the full structure of name‐based relationships to be visible. This search strategy enables broader retrieval of online content mentioning taxa (Beaudreau et al., 2011). We found that more culturally prominent taxa, such as elephants, and more generalized taxa, such as bats, tended to show higher content volume and wider geographic coverage across news and social media platforms. At the same time, this approach introduces polysemy because shared taxon names may appear in metaphorical, symbolic, or otherwise nonbiological contexts. This challenge is particularly acute for very general taxonomic groups or familiar taxa and necessitates scalable relevance filtering methods.

The use of general or culturally prominent taxon names in nonbiological contexts poses a recurring challenge for analyzing biodiversity discourse at scale. LLMs trained on general web content raise new possibilities for addressing such problems, particularly through zero‐shot approaches that apply these cutting‐edge models without task‐specific training. This removes the need for labeled data to train a model and allows these approaches to be applied with relatively limited effort, making them well suited to contexts with limited resources for model development. In our case, although precision varied across taxa, recall remained consistently high and the approach substantially reduced irrelevant content. For example, we found that up to 62% of articles mentioning bats were irrelevant, underscoring the importance of effective taxon‐relevance filtering. Further gains in precision could come from training a supervised relevance classifier, perhaps sampled across semantic space with the assistance of LDA topic outputs. After full‐text scraping, a second‐stage classifier could determine whether each occurrence of a taxon name is used in a biological sense, even in articles that include a mix of usages. These enhancements would support finer‐grained decisions but come at the cost of reduced flexibility and greater annotation effort.

Based on the data generated with our method, taxa differed in the distribution of topics in news media articles and in terms of their volume and sentiment through time in the news media or on Twitter. We observed a sharp increase in news coverage of horseshoe bats early in the COVID‐19 outbreak, contrasting with the other bat taxa in our case study, which exhibited no significant volume changes. Sentiment scores for articles and tweets mentioning horseshoe bats were generally positive but dropped earlier in the pandemic before rising again in late 2020, when we observed a significant breakpoint in news and Twitter discourse. Although the pandemic provided an illustrative case, our approach is designed for broader use. It can be applied to other major events, including conservation campaigns, policy announcements, and global biodiversity summits, to investigate how public discourse shifts around these moments. This includes examining whether public attention is sparked by such events or whether they arise within a landscape already shaped by growing public concern. These insights could help in the evaluation of the impact of public engagement initiatives and, over time, inform the design of more successful campaigns (Correia et al., 2021; Fernández‐Bellon & Kane, 2020; Hammond et al., 2022; Millard et al., 2021; Wright et al., 2020). Changes in coverage volume, or even a narrowing of topical focus around taxa, may signal early stages of societal extinction (Jarić et al., 2022), not only as species recede from public attention but also as the narratives surrounding them contract to limited frames. Tracking such patterns through automated analysis of coverage volume and sentiment offers a scalable, standardized way to monitor public interest in biodiversity more broadly (de Oliveira Caetano et al., 2022).

Our approach can serve as the foundation for an automated nature tracker that would help practitioners and researchers track public perceptions of biodiversity at scale. Monitoring human–nature perceptions is critical to evaluating progress toward the Global Biodiversity Framework targets, particularly those related to human–wildlife conflict and sustainable use. By scraping and processing data from news and social media, the system can deliver global, cost‐effective, near‐real‐time insights. To adapt the method for ongoing monitoring, scheduled queries (e.g., daily or hourly) to sources, such as GDELT, can feed directly into the existing pipeline, enabling continuous analysis without major structural changes. Therefore, digital approaches open new avenues for assessing compliance with the Global Biodiversity Framework, which is particularly pressing given that even as late as 2024 most of these targets still showed significant gaps in their evaluation mechanisms. Without effective tracking over substantial periods, conservation targets could be rendered politically irrelevant because it would be impossible to evaluate the progress (or lack thereof) made by different countries.

We have identified several key areas for future exploration and enhancement of our method. A primary aspect to address revolves around the linguistic scope of our approach, which currently centers solely on English‐language data. It will be key to broaden this scope to include other languages spoken in megadiverse countries, such as Spanish, Chinese, Portuguese, or Bahasa Indonesia. Furthermore, conservation social science monitoring must adapt to dynamic shifts in platform governance and data accessibility. Recent transitions in the ownership and management of Twitter underscore the urgency of this need. These transitions coincided with the proliferation of misinformation regarding climate change (King, 2023) and wildlife in the context of the COVID‐19 pandemic and a marked decline in active users, particularly environmentally focused users (Chang et al., 2023; Stokel‐Walker, 2022), both of which pose increasing challenges to monitoring approaches based on online data. As a step toward understanding how these dynamics shape public discourse about species and conservation, future work could incorporate NLP methods that detect misinformation—such as retrieval‐based fact checking (Guo et al., 2022)—and identify biased language, including emotive framing or hedging.

Overall, our study highlights the potential benefits of combining machine learning with the automated tracking of different data platforms to monitor public perceptions of biodiversity. We anticipate that methods such as ours or building on our approach can enhance applied conservation by creating new ways to examine human–nature perceptions at a global scale.

## Supporting information



Supporting Information
