# Supplementary material for: Automating the analysis of public saliency and attitudes toward biodiversity from digital media
Source: Conserv Biol. 2026 Jan 18;40(2):e70217. doi: 10.1111/cobi.70217 (PMC13036311; doi:10.1111/cobi.70217)
Supplement: Supplementary file 1 — Supporting Information [file COBI-40-e70217-s001.docx]

Supporting Information

## Appendix S1

**Folk Taxonomy Graph Construction**

To construct the graph, a node is created for every:

- scientific name for a species (e.g. *Rhinolophus affinis*)
- full common name for a species (e.g. “Intermediate horseshoe bat”)
- substring shared between multiple common names (e.g. “Horseshoe bat” is part of “Intermediate horseshoe bat” and “Greater horseshoe bat”) or substrings (e.g. “Bat” is part of “Horseshoe bat” and “Fruit bat”)

Edges exist between the nodes representing the scientific name and the full common names for each species, as well as between common names and their substrings.

## Appendix S2

Folk taxonomy entries selected for this study are listed, with positive keywords derived from our hierarchical graph construction on common name endings and negative keywords obtained by pruning edges that connected disparate taxa. The focal taxa ranged from the genus level, such as *gorilla*, to the order level, such as *bat*. They included more specific yet popularly recognized taxa like *flying fox* and *vampire bat*, as well as lesser known but distinct taxa like *pipistrelle* and *horseshoe bat*.

Table mapping focal folk taxa used in this study to their corresponding scientific taxa and keyword sets.

| **Order** | **Scientific taxon** | **Folk taxon** | **Positive keywords** | **Negative keywords** |
| --- | --- | --- | --- | --- |
| Chiroptera | Order Chiroptera | Bat | bat |  |
| Chiroptera | Family Pteropodidae | Flying fox | flying fox, pale xantharpy, acerodon, monkey-faced bat, greater nectar bat, fruit bat, north moluccan blossum-bat, rousette, golden bat of rodrigues, codot horsfield, woerman’s bat, blossom bat | tube-nosed fruit bat |
| Chiroptera | Family Hipposideridae, Family Rhinonlophidae | Horseshoe bat | diadem leafnosed-bat,roundleaf bat, horseshoe-bat, horseshoe bat, great woolly horseshoe bat, trident bat, leaf-nosed bat, flower-faced bat |  |
| Chiroptera | Genus Glossophaga,  Genus Craseonycteris,  Genus Leptonycteris | Long tongued bat | hog-nosed bat, long-nosed bat, bumblebee bat, long-tongued bat |  |
| Chiroptera | Genus Myotis, Genus  Perimyotis | Myotis | pond bat, bocage’s banana bat, van hasselts bat, social bat, bechstein’s bat, hodgson’s bat, lesser large-tooth bat, mouse-eared bat, ridley’s bat, water bat, ikonnikov’s bat, whiskered bat, welwitch’s bat, three-coloured bat, daubenton’s bat, indiana bat, fish-eating bat, grey bat, siliguri bat, geoffroy’s bat, large-footed bat, myotis, horsfield’s bat, rickett’s big-footed bat, little brown bat, brandt’s bat, siberian bat, morris’s bat, natterer’s bat, intermediate bat, hairy-faced bat, Descaleras bat |  |
| Chiroptera | Subfamily Vespertilioninae | Pipistrelle | pipistrelle, pipistrelle bat, cape bat, anchieta’s bat, rohus bat, thai golden-throated bat, siam goldnecklet, dormer’s bat, ruppell’s bat, rusty bat, little indian bat, white-winged bat, sind bat, aloe bat, hottentot bat, indian pygmy bat, banana bat | bocages banana bat |
| Chiroptera | Family Emballonuridae, Family Phyllostomidae, Family Megadermatidae | Vampire bat | ghost bat, vampire bat, false vampire, heart-nosed bat |  |
| Proboscidea | Family Elephantidae | Elephant | elephant |  |
| Pholidota | Family Manidae | Pangolin | pangolin |  |
| Primates | Genus Gorilla | Gorilla | gorilla |  |

## Appendix S3

News articles for each focal taxon were classified as biologically relevant or irrelevant based on topic predictions from their titles. Article full text was scraped and used to determine whether each article was original or syndicated through text-similarity analysis.

The counts across taxa for original versus duplicate articles identified in our method and relevant articles determined by zero-shot learning with a transformer model.

|  | **Predicted relevance:** | **True** | **False** |
| --- | --- | --- | --- |
| **Taxon** | **Original article** |  |  |
| Elephant | True | 86425 | 70548 |
|  | False | 69335 | 54403 |
| Gorilla | True | 21399 | 21206 |
|  | False | 11715 | 9476 |
| Pangolin | True | 6286 | 1495 |
|  | False | 5976 | 1055 |
| Bat | True | 77802 | 129058 |
|  | False | 45452 | 68122 |
| Flying fox | True | 1969 | 601 |
|  | False | 1311 | 447 |
| Myotis | True | 1103 | 103 |
|  | False | 978 | 21 |
| Horseshoe bat | True | 791 | 171 |
|  | False | 745 | 257 |
| Pipistrelle | True | 496 | 63 |
|  | False | 489 | 45 |
| Vampire bat | True | 341 | 195 |
|  | False | 357 | 176 |
| Long-tongued bat | True | 150 | 24 |
|  | False | 79 | 9 |

## Appendix S4

Counts of X (Twitter) posts from 1 January 2019 to 31 December 2021 for all 10 focal taxa analyzed in this study.

| **Entity** | **Count** |
| --- | --- |
| Elephant | 171059 |
| Gorilla | 52650 |
| Pangolin | 3667 |
| Bat | 325319 |
| Flying fox | 1889 |
| Myotis | 166 |
| Horseshoe bat | 140 |
| Pipistrelle | 354 |
| Vampire bat | 462 |
| Long-tongued bat | 80 |

## Appendix S5

Chord diagrams depicting the co-occurrence of topics in relevant news articles for all 10 focal taxa analyzed in this study (arcs, topics occurring together in articles; wider the arc, the more articles that contained a given topic pair; colors, different groups of topics; sections on the perimeter of circles, proportional occurrence of each topic [e.g., sports] in the dataset).

## Appendix S6


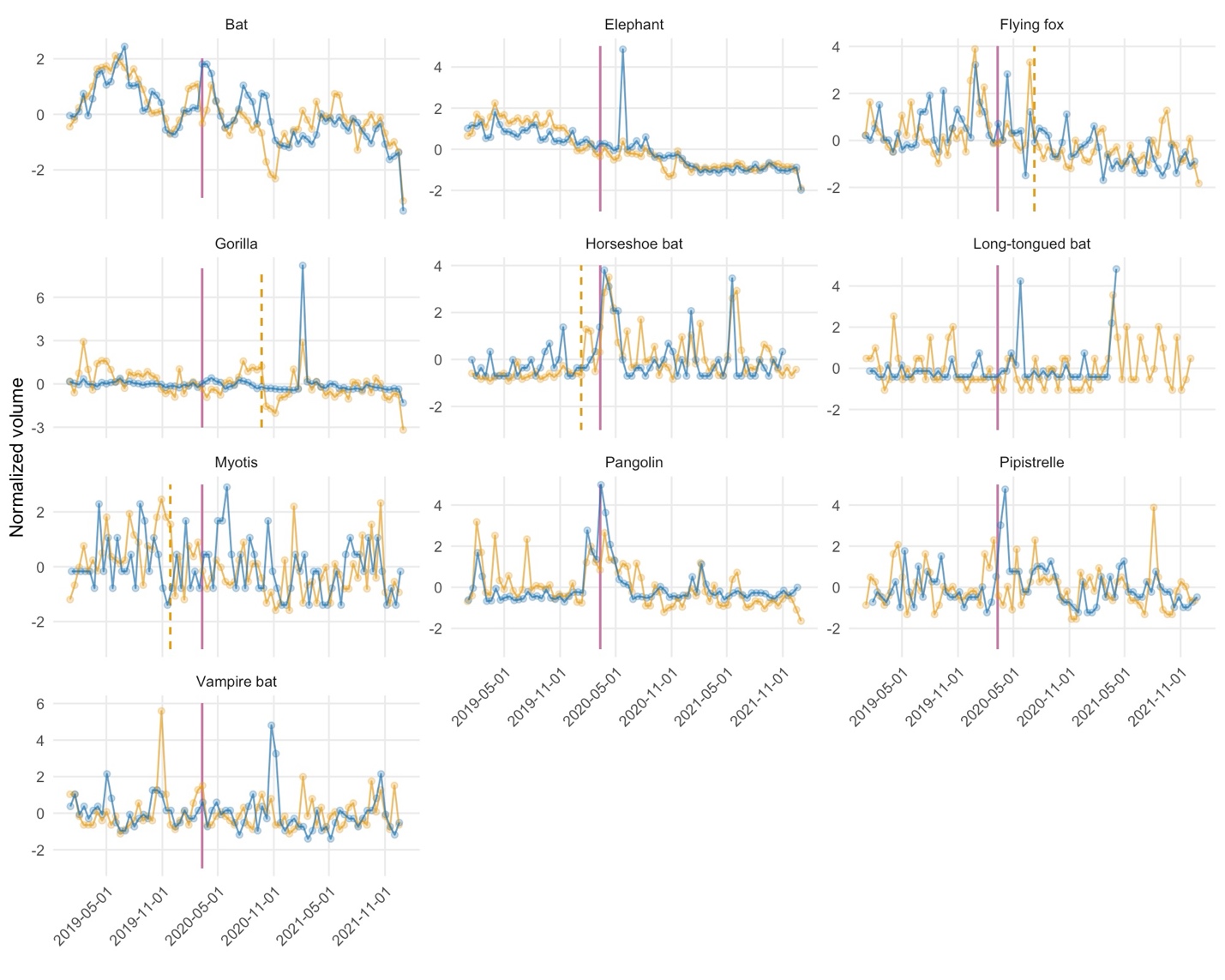


Z-score-normalized time series of the number of news articles and social media posts mentioning each of the 10 focal taxa analyzed in this study (gold lines, news; blue lines, social media; vertical magenta line, the World Health Organization’s COVID-19 pandemic declaration on 11 March 2020; dashed vertical gold lines, Bonferroni- corrected significant breakpoints in the trends for news).

## Appendix S7


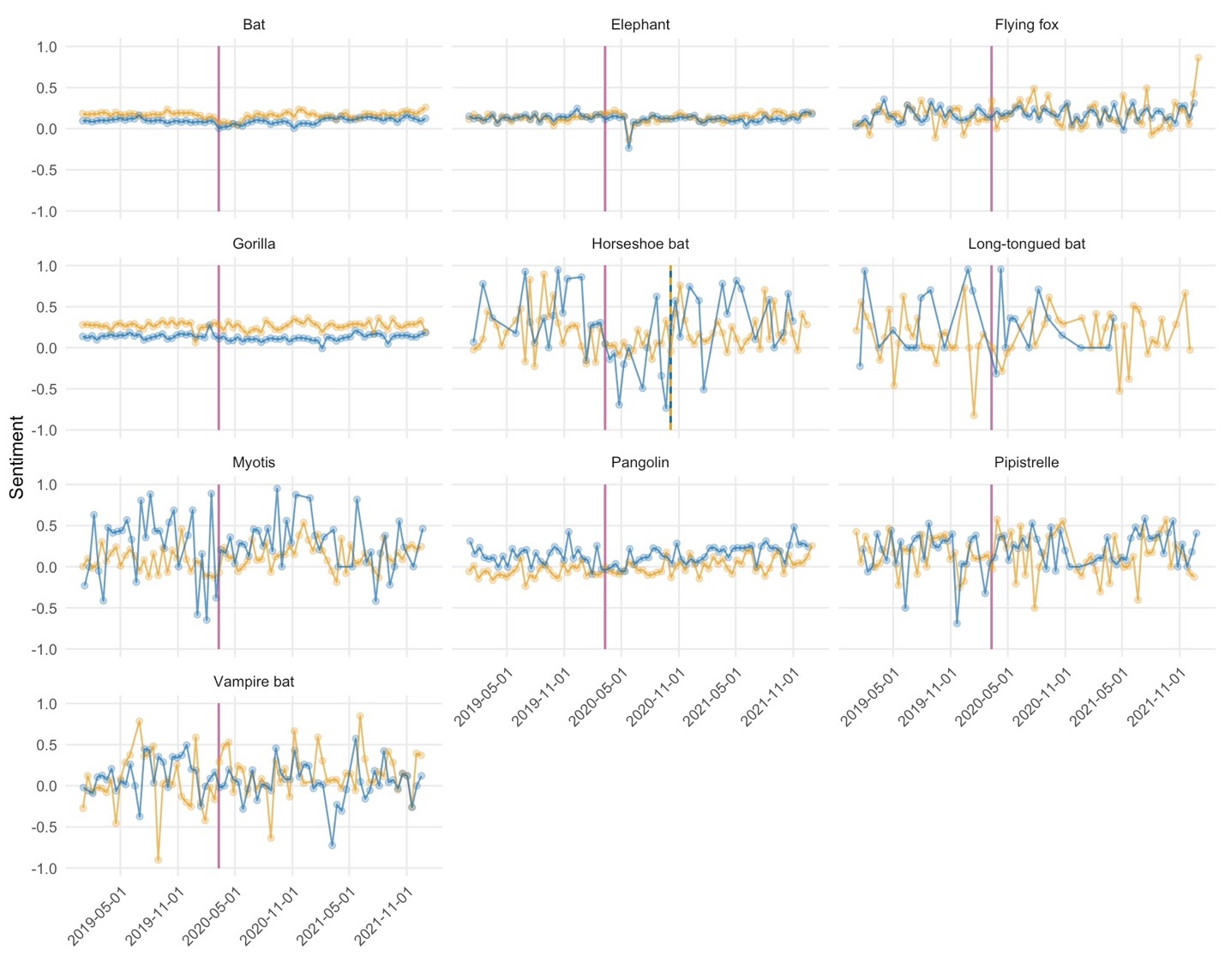


Time series of the sentiment of news articles and social media posts mentioning each of the 10 focal taxa analyzed in this study (gold lines, news; blue lines, social media; vertical magenta line, the World Health Organization’s COVID-19 pandemic declaration on 11 March 2020; dashed vertical gold or blue lines, Bonferroni- corrected significant breakpoints in the trends for news or social media respectively).
